# Estimating epidemiological parameters for bovine tuberculosis in British cattle using a Bayesian partial-likelihood approach

**DOI:** 10.1098/rspb.2014.0248

**Published:** 2014-05-22

**Authors:** A. O'Hare, R. J. Orton, P. R. Bessell, R. R. Kao

**Affiliations:** 1 Boyd Orr Centre for Population and Ecosystem Health, Institute of Biodiversity, Animal Health and Comparative Medicine, College of Medical, Veterinary and Life Sciences University of Glasgow, Glasgow G61 1QH, UK; 2 The Roslin Institute, The University of Edinburgh, Easter Bush, Edinburgh, EH25 9RG, UK

**Keywords:** partial likelihood, ergodic, bootstrap, nonlinear dynamics

## Abstract

Fitting models with Bayesian likelihood-based parameter inference is becoming increasingly important in infectious disease epidemiology. Detailed datasets present the opportunity to identify subsets of these data that capture important characteristics of the underlying epidemiology. One such dataset describes the epidemic of bovine tuberculosis (bTB) in British cattle, which is also an important exemplar of a disease with a wildlife reservoir (the Eurasian badger). Here, we evaluate a set of nested dynamic models of bTB transmission, including individual- and herd-level transmission heterogeneity and assuming minimal prior knowledge of the transmission and diagnostic test parameters. We performed a likelihood-based bootstrapping operation on the model to infer parameters based only on the recorded numbers of cattle testing positive for bTB at the start of each herd outbreak considering high- and low-risk areas separately. Models without herd heterogeneity are preferred in both areas though there is some evidence for super-spreading cattle. Similar to previous studies, we found low test sensitivities and high within-herd basic reproduction numbers (*R*_0_), suggesting that there may be many unobserved infections in cattle, even though the current testing regime is sufficient to control within-herd epidemics in most cases. Compared with other, more data-heavy approaches, the summary data used in our approach are easily collected, making our approach attractive for other systems.

## 1. Introduction

Infectious diseases with long generation times are challenging to model, owing to the uncertainty in identifying the patterns of infectious contacts, a problem that can be exacerbated by the influence of a wildlife reservoir host. For bovine tuberculosis (bTB) in Great Britain (GB), both badgers and cattle contribute to the epidemiology [1,2], and despite an exceptional record of the history of the disease in cattle, the relative roles of the two host species remain controversial. As modelling the cattle data in their entirety is a considerable computational challenge, identifying what data are important for understanding the epidemiology is essential for allowing greater sophistication in model structure, with the availability of extensive data allowing for comparisons with models that exploit the data more completely.

The single intradermal comparative cervical tuberculin (SICCT or ‘skin’ test) test is the standard test for all ante mortem testing in GB. It measures a delayed type hypersensitivity response to intradermally injected tuberculin [3]. Prior to 2013, herds in GB were routinely tested (routine herd test; RHT) at intervals of 1–4 years, based on local historical prevalence. One or more confirmed reactor (i.e. positive SICCT test) results in a herd breakdown (now ‘officially Tb-free withdrawn’ or OTFW) and is subjected to movement restrictions and follow-up tests. Inconclusive tests leads to follow-up testing (OTF suspended if the herd had OTFW within the past 3 years) until cleared or confirmation is made. Once OTFW, all reactors are culled, and restrictions continue until the herd passes two successive clear tests not less than 60 days apart [4] with all tests being interpreted under ‘severe’ criteria that increase test sensitivity, but at the cost of additional false-positives. In addition, all cattle more than 41 days old moving from a 1 or 2 yearly tested herd are subjected to additional testing requirements [5]. All cattle sent to slaughter are subjected to a post-mortem inspection. bTB suspect lesions result in samples being sent for culture and isolation of the causative bacteria *Mycobacterium bovis*, confirmation leads to a breakdown/OTFW, and a whole herd test (WHT) is applied. Additional testing occurs based on forwards and backwards contact tracing from identified breakdowns. Current sensitivity estimates are low for the SICCT test (approx. 50%) under the ‘standard interpretation’, and for post-mortem inspection (approx. 70%) [6]. Both are considered to have very high specificity.

Diseases such as bTB have long, poorly quantified stages of disease progression, with estimates of a latent or exposed period, of 0–63 days [3,7–9] and 180 *±* 20 days for a test-sensitive stage where infectivity is low, but SICCT test detection is possible [10].

To better quantify such disease parameters, model fitting using Bayesian likelihood-based approaches are becoming increasingly important in infectious disease epidemiology, and have shown particular promise in systems with detailed population characteristics [11–13]. Previous use of these approaches for bTB in cattle used detailed longitudinal life-history data for cattle, and also required either the use of approximate Bayesian computation (ABC) [14], or are computationally tractable only for small datasets [15].

Here, we adapt a previous model [16] as the basis for a less data-heavy approach to inference; although explicit account is taken of the herd age structure and testing schedules in GB, we use a likelihood function based only on the number of reactors at first identification to estimate key epidemiological parameters.

## 2. Model formulation

**Figure 1.**
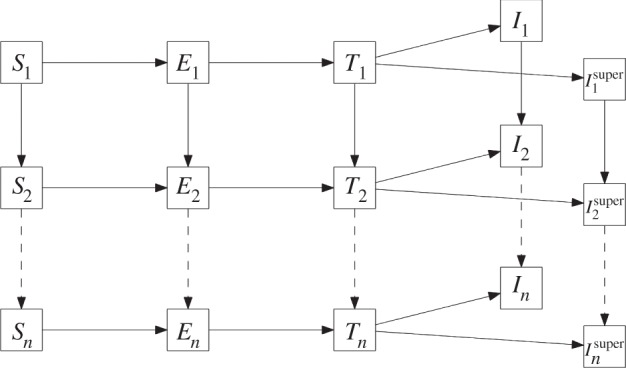
Disease propagation though the infection stages, *susceptible*, *exposed*, *test-sensitive* and *infectious* in the age-based SETI model. Individuals move either into a new infection stage (horizontally) or to the next age bracket (vertically) as denoted by subscripts. There are two classes of infectious individuals; super-spreaders (when included in the model) are categorized as super-infectious where the transmission parameter is scaled by the value *ζ*.

We consider a hierarchy of nested model structures, where, in the simplest case, cattle are either *susceptible*, *exposed*, *test-sensitive* or *infectious*. Once an animal becomes infectious, it remains so until it is detected, at which point the animal would be culled. Each compartment is further split into *N_A_* separate age groups. Susceptible cattle become *exposed* through infectious contact within the herd, and through external factors that may include for example, inward cattle movements, contiguous spread from neighbouring herds or the presence of a wildlife reservoir. These external factors are incorporated into the model via a single force of infection. The model is depicted schematically in figure 1.

We allow for heterogeneity in the infectiousness of individuals by incorporating ‘super-spreaders’, i.e. with some individuals more likely than average to infect others if, for example, it excretes more bacteria than average, as suggested by experimental data [17]. A fraction of the herd, *P*_S_, are modelled as super-spreaders, where the transmission term is scaled by a factor *ζ*_S_.

The average infectiousness of individuals may vary between herds [18], and this is incorporated into the model by allowing transmission of the disease for all livestock to be scaled by a factor *ζ*_H_ in a proportion of herds, *P*_H_, and similarly we consider two levels of variability in *α*, the transmission rate from the reservoir.

The deterministic model is written as a system of ordinary differential equations:2.1
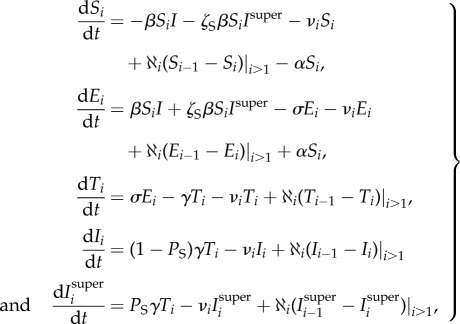
where subscripts denote the age group, infection states with no subscript means the sum over all groups, e.g. *I* = ∑*_i_I_i_*, and *α* is the force of infection external to the herd. The parameter 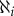 is the rate cattle in one age group move up to the next (older) age group, so that 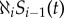 is the number of cattle entering the *S_i_*(*t*) state and 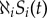 the number leaving. Cattle in the oldest age group are removed from the system at the rate 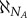. Cattle are also removed from the herd (via death or export) at a rate *ν_i_*, where we allow for age-specific removal rates giving *ν_i_S_i_*(*t*) as the number of susceptible cattle in the *i*th age group being removed from the herd at time *t*. In each simulated epidemic, we assume a constant herd size, assuming replacements are drawn from the same age distribution. We make the simplifying assumption that replacements are all susceptible; in high-risk areas (HRAs), the effect of having some infectious replacements is subsumed in *α*, whereas in low-risk areas (LRAs), where movements from HRAs are few, the likelihood of multiple introduction is low owing to the low overall prevalence of infection in all cattle (of 5 417 573 tests carried out in 2006, only 20 090 confirmed reactors were found [19]).

**Figure 2.**
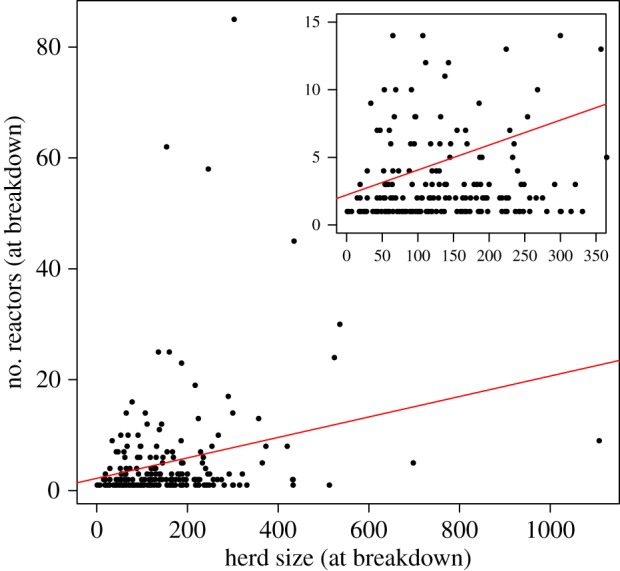
Number of reactors detected to the herd size at breakdown as a function of herd size (inset shows a detailed view of small herds). The correlation is weak but suggests some density dependence (Spearman's *ρ*-value of 0.277). (Online version in colour.)

As herd size is known to be correlated to infection persistence [20] (figure 2), we assume density-dependent transmission, with infection occurring at base rate *βIS*. Exposed cattle become test-sensitive at a rate *σ* and then infectious at a rate *γ*.

Variability in *ζ*_H_ and *α* are implicit as herd heterogeneity is determined before each individual simulation with fitted probabilities.

### (a) Initialization

We assume that outbreaks are initially seeded by a single randomly chosen infected animal. Similar to others [14], we assume that the occurrence of breakdowns owing to the introduction of multiple infections is low; this probably causes a compensatory increase in the estimated transmission rate, but we expect this effect to be small (see above on national prevalence).

### (b) Process overview

The model (2.1) was solved by running 2 × 10^4^ independent simulations using Gillespie's *τ*-leap method with a fixed time step of 14 days to balance simulation efficiency and model accuracy. We used Gillespie's direct method [21] to validate the choice of time step in the *τ*-leap method. In each simulation, herd size and age structure are selected from the observed distributions in GB and run to the random predetermined future date selected from a uniform distribution over 0 to *n* years, where *n* is the testing interval. We then perform an RHT with test sensitivity *Ω*_r_. The number of reactors at the time of a test, *N_B_*, is therefore2.2
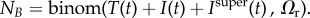


If no infected cattle are detected, then we schedule another RHT *n* years later and continue running the simulation. If, at any time, an animal is removed from the herd, then it is subjected to a post-mortem test with net sensitivity *Ω*_s_, considering the combined probability of being inspected and detected. A positive test triggers a breakdown resulting in a WHT. Any confirmed breakdown sets the RHT schedule to every two months until there are no further reactors. When a breakdown is detected (by either RHT or at abattoir), the number of reactors is added to a frequency distribution for routine and triggered WHT tests from which we compare the distribution with the number of reactors at first detection as recorded in VetNet. Each simulation is run for a maximum of 20 years.

The frequency distribution of reactors at first breakdown is interpreted as a multinomial trial with *p*_1_, *p*_2_, …, *p_n_*, the probability of the number of reactors being detected as 1, 2, …, *n*, and *x*_1_, *x*_2_, …, *x_n_*, the number of times we detected 1, 2, …, *n* reactors in the herd at breakdown in the simulation. Using the observed (age-independent) breakdown size distribution, we calculate the probabilities *p*_1_, *p*_2_, …, *p_n_* for both the breakdowns detected from routine RHT and abattoir-triggered WHT giving a likelihood function2.3
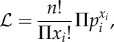
where *n* is the total number of breakdowns observed in the simulation and *x_i_* are the frequencies of detecting each breakdown size in our simulations. The free parameters in the described model are *β*, *σ*, *γ*, *Ω*_r_, *Ω*_s_, *P*_S_, *P*_H_, *ζ*_S_, *ζ*_H_, *α*. We calculate the basic reproduction number, *R*_0_, according to the ‘next-generation matrix’ approach defined by Diekmann *et al*. [22] for each parameter set in the posterior.

We use the Metropolis–Hastings algorithm [23] to generate parameter posterior distributions. Each trial step is determined by selecting each parameter from a normal distribution whose mean is the parameter value of the current step and a fixed standard deviation of 2% of the mean. We decrease the variance by 10% after every 2000 steps. We perform several random walks (chains) for each model starting at different points in parameter space and where each chain has a length of 10 000 steps. The posterior distribution is determined by removing the ‘burn-in’ from the chains. The model was coded in Java. The inference scheme was tested for self-consistency by running simulated epidemics using the model structures as defined above and using our inference approach to estimate the model parameters. These were shown to recover the input parameters with posterior distributions of similar width to those identified for our real data, and with the original parameters lying within the 95% credible intervals of the posteriors (results not shown).

### (c) Input

Cattle test data were obtained from the VetNet and Vebus databases obtained from the Animal Health and Veterinary Laboratories Agency (AHVLA). The cattle distribution throughout GB was obtained from the cattle tracing system database from the Department for Environment, Food and Rural Affairs (DEFRA). We use RHT and WHT records from 2006, because they were sufficiently long after the 2001 foot and mouth disease outbreak for the resultant perturbations in bTB incidence to have disappeared and because quadrennial testing areas expanded rapidly before that year. This dataset was filtered to consider only breakdowns triggered by RHTs or through tracing from abattoir detection in parishes exclusively tested annually (long-term HRAs) or quadrennially (long-term LRAs) from 1998 to 2006. In addition, we consider only breakdowns where there had not been a previous positive test in the herd and do not consider the results of follow-up tests; this minimizes the impact of possible infections missed from previous outbreaks, and mitigates against possibly epidemiologically significant changes in farmer behaviour after an outbreak begins. This leaves 1533 incidents with 4498 reactors in HRAs, and 78 incidents with 138 reactors in LRAs. Only herd breakdowns with confirmed reactors (i.e. where visible lesions have been identified) are included; unconfirmed reactors are statistically more likely so show up as reactors later [24]. Inconclusive unconfirmed reactors that never test positive are excluded from the analysis; although there is a statistically significant risk of transmission indirectly associated with them, the absolute risk is slight [25], and therefore likely to be dominated by the transmission from confirmed reactors.

The distribution of the number of reactors at first breakdown is recorded then defines the likelihood function (equation (2.3)). We calculate the distribution of herd sizes based on the recorded number of cattle when it first suffered a breakdown (i.e. using the same criteria used to obtain the breakdown size distribution to define our likelihood). We use the age structure as found in VetNet, the age distribution of reactors and the age distribution of cattle sent to slaughter, identifying 14 age groups with lengths from two to 24 months. Uniformly distributed priors based on field and experimental data were used where found (table 1) [6,26] with non-informative priors where no estimates existed.

**Table 1.** Summary of the priors used in the model.

parameter	description	sampling distribution
*β*	transmission rate	uniform (1 × 10*^−^*^5^, 1 × 10*^−^*^2^)
*σ*	rate of exposed cattle becoming test-sensitive	uniform (6 h−100 days)
*γ*	rate of test-sensitive cattle becoming infectious	uniform (four to nine months)
*Ω*_r_	probability that a test-sensitive or infectious animal is detected by the SICCT test	uniform (40−80%)
*Ω*_s_	probability that a test-sensitive or infectious animal is detected at abattoir	uniform (50−99%)
*ζ*_S_	increased infectiousness of super-spreaders	uniform (1, 1000)
*P*_S_	proportion of individuals that are super-spreaders	uniform (1 × 10*^−^*^3^, 0.4)
*ζ*_H_	increase of *β* in herds with high *β*	uniform (1, 1000)
*P*_H_	proportion of herds with high *β*	uniform (1 × 10*^−^*^3^, 0.4)
*α*	external force of infection	uniform (1 × 10*^−^*^10^, 5 × 10*^−^*^3^)

For *σ*, *γ*, *Ω*_r_, *Ω*_s_, the priors were chosen on the basis of existing field and experimental estimates [6,26], non-informative priors were used for all other parameters. The rate-exposed cattle become test-sensitive, and test-sensitive cattle become infectious is the inverse of the exposed and test-sensitive periods (6 h−100 days and four to nine months), respectively.

## 3. Results

Multiple chains were run for each model, and only chains reaching the same posterior-likelihood distribution (as measured by the Gelman–Rubin statistic [27]) were retained. Some multi-modal behaviour in parameter posteriors was observed which is the result of correlations between the parameters in the model (confirmed using principal component analysis and discussed further in the electronic supplementary material).

**Figure 3.**
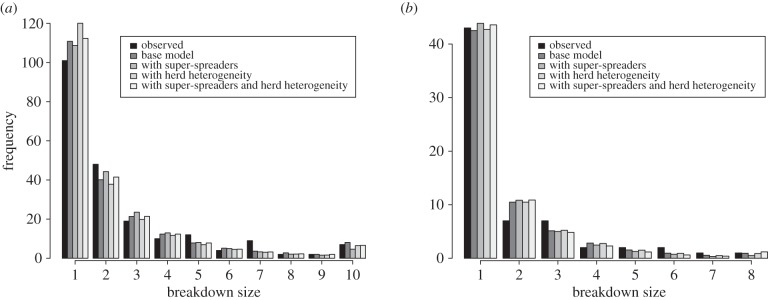
Comparison of the distribution of breakdown sizes for the models investigated for HRAs (top) and LRAs (bottom). The number of reactors found when a herd breaks down is determined over several simulations for each model and the distribution converted to a likelihood value that is used in the Markov chain Monte Carlo chain. We have grouped those breakdowns with 10+ reactors in HRA and 8+ reactors in LRA together.

The distribution of the number of reactors in the herd at first detection for all models is shown in figure 3. The maximum-likelihoods were compared using Akaike information criterion (AIC) scores (table 2) for purposes of model selection. In LRAs, no heterogeneity in transmission is favoured, whereas in HRAs, there is a slight improvement in AIC for the ‘super-spreader’ model. Both were substantially better than models with herd heterogeneity.

**Table 2.** AIC scores for the models. (Individual level heterogeneity provides a small improvement over a model without heterogeneity in high-risk areas (annually tested) but no such heterogeneity is required for low-risk areas (quadrennially tested).)

model	AIC score
1 year	61.8
1 year with individual transmission heterogeneity	60.6
1 year with herd transmission heterogeneity	64.3
1 year with individual and herd transmission heterogeneity	65.3
1 year with individual and reservoir heterogeneity	75.8
1 year with reservoir heterogeneity	86.6
4 year	33.0
4 year with individual transmission heterogeneity	37.7
4 year with herd transmission heterogeneity	37.5
4 year with individual and herd transmission heterogeneity	41.4

**Figure 4.**
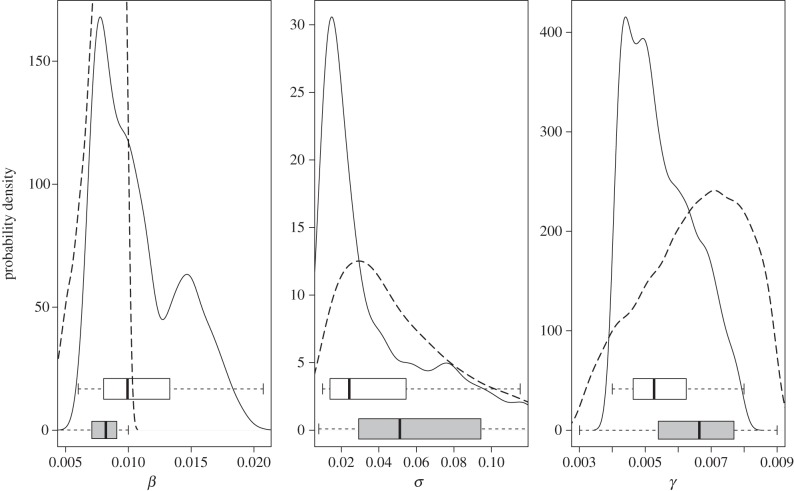
Comparison of the posterior distributions of transmission parameters in this model with an independent approach [15]. The solid and dashed lines and (open and shaded) box-plots denote those distributions for Great Britain and Northern Ireland, respectively, where *β* is the transmission parameter, *σ* is the rate exposed cattle become sensitive to the SICCT test, and *γ* is the rate cattle move from the test-sensitive class to the infectious class.

Posterior distributions for comparable epidemiological parameters for this model and one using detailed cattle life histories [15] are notably similar (figure 4). Although other parameters cannot be directly compared owing to differences in model and data structures, the two approaches also give similar estimates of *R*_0_ (1.3−1.9 in HRAs and 0.6−1.4 in LRAs).

In HRAs, the length of the exposed stage (i.e. 1/*σ*) was estimated to be approximately 100 days (with lower and upper quartiles 14–100 days), whereas in LRAs, it was estimated to be lower at approximately 60 h (lower and upper quartiles 28 h–10 days). These differences do not appear to be due to the differences in the mechanism of introduction, for example, if introduction in HRAs is more likely to be owing to infection of resident cattle and in LRAs owing to movement of already infected and potentially infectious cattle (see the electronic supplementary material for a test of this). Both estimates do overlap with previously published data [3,7–9,15].

**Figure 5.**
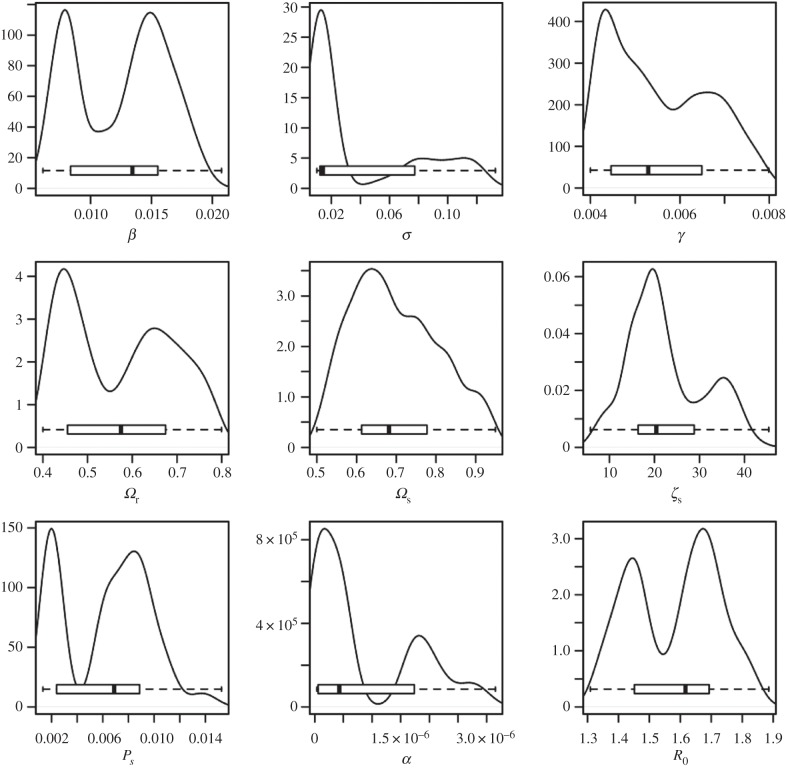
Posterior kernel density estimates for the parameter distributions for the preferred model (including super-spreaders) for annually tested areas. Here *β*, *σ*, *γ* are the transition rates in our model, *Ω*_r_, *Ω*_s_ are the sensitivities of the routine SICCT and abattoir tests. Individual super-spreader transmission is scaled up by *ζ*_S_ with *P*_S_ the probability of an individual being a super-spreader. The external force of infection is denoted by *α*.

The length of the test-sensitive stage (i.e. 1/*γ*) in HRAs was estimated to be approximately 190 days (with lower and upper quartiles 150 and 220 days, respectively) and in LRAs estimated to be approximately 180 days (with lower and upper quartiles of 150 and 200 days). The estimates for this stage also agree with previously published estimates of 180 days ±20 days [10,14,15] (figure 5).

**Figure 6.**
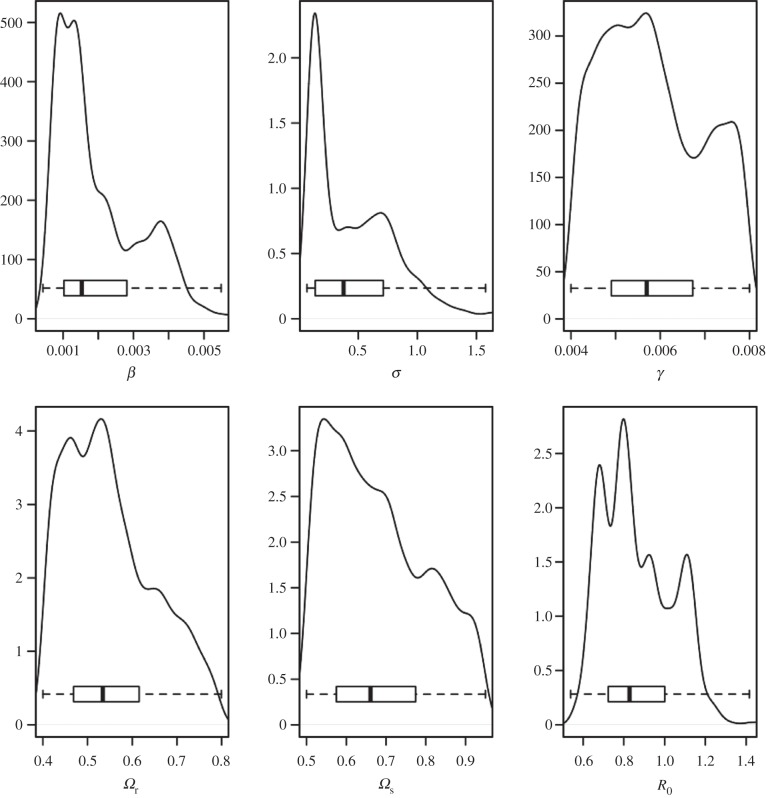
Posterior kernel density estimates for the parameter distributions for the preferred model (including neither individual super-spreaders nor super-spread herds) for 4 year testing areas. Here *β*, *σ*, *γ* are the transition rates in our model, *Ω*_r_, *Ω*_s_ are the sensitivities of the routine and abattoir tests.

Estimates for the sensitivities of the SICCT tests are consistent with previous observations [6,8,26] in both annual and quadrennial year testing areas with a mean value of approximately 55% (with quartile range of approx. 45% and approx. 65%). The posterior distribution for the sensitivity of the abattoir tests is approximately 67% (quartile range 60–80%) consistent with the 70% value suggested by Downs *et al*. [6] (figure 6).

In both HRAs and LRAs, there may be a considerable time before infection is detected. After breakdown, many undetected, infected cattle may remain in the herd. Our simulations show that only approximately 45% of detectable infection is detected within 12 months of infection (i.e. by the first test after introduction) in annual testing areas and approximately 60% is detected in quadrennial testing areas within 48 months. In both areas, approximately 25% are missed by routine surveillance. Once a herd is OTFW, it is subjected to movement restriction and follow-up tests until the herd is deemed clear of infection. The duration of these restrictions or ‘episode length’ was calculated in our model and compared with the data held in the national testing database. Our simulations show a consistency between HRA and LRAs with approximately 80% of episodes lasting 12 months or less. The longest recorded outbreak is shorter than approximately 30% of simulated epidemics; this may be owing to changes in farm management post-breakdown that are not reflected in the model, but are effective in reducing the overall episode length.

The posterior estimate for *α*, the external force of infection, is approximately 5 × 10*^−^*^7^ new infections per susceptible animal per day. This rate is lower than the cattle-to-cattle transmission; however, the overall impact of *α* remains high, as *α* is active over the entire residence time of a susceptible animal in HRAs, whereas an infectious animal is only active over its infectious period until removal owing to death or export. Thus, both internal and external factors appear to be important in driving the observed breakdown patterns.

The 95% credible intervals for the posterior predictive estimates for *R*_0_ in HRAs and LRAs are 1.3−1.9 and 0.6−1.4, respectively. This is similar to previous estimates of *R*_0_, but as it directly incorporates the distribution of herd sizes in each area, differs from the estimates of high *R*_0_ for large herds found in a previous analysis [14]. This difference may be due to our consideration of a timeframe after the rapid expansion of annual testing areas (see the electronic supplementary material, figure S2), and bears further investigation.

## 4. Discussion

Our fitting approach derives from the principle of ergodicity, i.e. the distribution of outcomes across multiple outbreaks provides the same information as the distribution of observations of a single system if we could observe it over all time. It effectively assumes a stationary outbreak distribution even though the national epidemic itself is expanding. Despite the individual differences between herds, our comparison shows that there is sufficient information in this one statistic to reproduce key outputs consistent with models that use more detailed outbreak data. This approach also has some advantages over ABC, where model selection has known technical challenges [28]; here, the calculation of an explicit likelihood simplifies model selection, albeit at the cost of a simplified comparison.

Our analysis shows that, broadly speaking, in both areas simple models fit well, with only a slight preference for greater heterogeneity in HRAs and herd-level heterogeneity strongly rejected. These small differences imply that our model selection outcomes are indicative only rather than conclusive statements in themselves. Differences in the transmission rates associated with super-spreaders do suggest that, should these results be supported by further evidence, there may be considerable value in identifying these individuals in controlling the disease.

The relative efficacy of abattoir inspection and routine testing has previously been directly estimated using more extensive data on the time course of the epidemic [14], and our estimates using a more compact summary of the data are similar. While our estimate of net abattoir testing sensitivity is based both on the probability of inspection (i.e. proportion of removed cattle moving to slaughter) and detection, it is likely that it is dominated by the latter—the most common cattle life history involves direct move to slaughter from the birth premises [29,30] and of those that move more frequently, many are younger animals moving to low-risk finishing units [31]. Significant savings can be made by reducing the extent of routine herd testing in LRAs as the risk of onward transmission is low, and therefore missed infections are likely to have little impact, consistent with other findings [26]. However, while the sensitivity of the SICCT test is poor, it is likely to result in more rapid identification of breakdown herds, reducing the risk of onward transmission. The impact of rapid identification on between herd transmission must be explored more thoroughly, especially in HRAs. Despite dramatic differences across the model tested, estimates of the sensitivities of testing and the role of external infections remains broadly and encouragingly similar, suggesting that further model refinements coming at higher computational cost are unlikely to change our estimates of these important parameters.

While it is not possible to attribute the source of the external force of infection based on the model alone, badgers are likely to be at least partially involved. At introduction, in most herds, the force of infection owing to external causes is considerably lower than the within-herd force of infection suggesting cattle-to-cattle transmission is usually dominant. Previous low estimates for the role of interherd transmission in sustaining the national epidemic [32] support the view that only a few herds are responsible for onward transmission to LRAs, and a self-sustaining cattle epidemic unlikely. Thus, the balance of internal and external factors would suggest that any control programme must consider both mammalian hosts in order to succeed. These, however, are better addressed by integrated models that consider both within-herd and between-herd transmission.

In this paper, we used a constant value for the external force of infection, because the outbreak size has a weak dependence only on the size of the herd (figure 2). However, external factors may also vary with herd size; for example, nose-to-nose contact with other herds may also increase with fence length, as might the total grazing area and therefore potential exposure to infected badgers. Exploring these relationships could be done through examination of land-use data as has been done for foot and mouth disease [33] and badger density estimates [34]. Another consideration that stands out is the difference in exposed period across the two areas: one possible explanation may be that differences in infecting route and dose influence the duration of the exposed state.

Simple models such as presented here are of course a caricature of the true epidemiological situation; individual herds will vary in structure and composition, and explicit herd outbreak histories could be exploited in more detailed studies. Despite the partial nature of the likelihood that we adopt, we have shown that it captures essential elements of the transmission process. As such data as we use here (outbreak size and age structure) are much more likely to be collected for other disease systems, the approach outlined in this paper has the potential for application across a wide range of infectious diseases.
